# The economic burden of influenza among adults aged 18 to 64: A systematic literature review

**DOI:** 10.1111/irv.12963

**Published:** 2022-02-05

**Authors:** Caroline de Courville, Sarah M. Cadarette, Erika Wissinger, Fabián P. Alvarez

**Affiliations:** ^1^ Global Health Economics and Value Assessment Sanofi Pasteur Lyon France; ^2^ Evidence Synthesis & Modeling Xcenda, L.L.C. Carrollton Texas USA

**Keywords:** adult, cost of illness, human, influenza, working adults

## Abstract

While the economic burden of influenza infection is well described among adults aged 65 and older, less is known about younger adults. A systematic literature review was conducted to describe the economic burden of seasonal influenza in adults aged 18 to 64 years, to identify the main determinants of direct and indirect costs, and to highlight any gaps in the existing published evidence. MEDLINE and Embase were searched from 2007 to February 7, 2020, for studies reporting primary influenza‐related cost data (direct or indirect) or absenteeism data. Of the 2613 publications screened, 51 studies were included in this review. Half of them were conducted in the United States, and 71% of them described patients with influenza‐like illness rather than laboratory‐confirmed disease. Only 12 studies reported cost data specifically for at‐risk populations. Extracted data highlighted that within the 18‐ to 64‐year‐old group, up to 88% of the economic burden of influenza was attributable to indirect costs, and up to 75% of overall direct costs were attributable to hospitalizations. Furthermore, within the 18‐ to 64‐year‐old group, influenza‐related costs increased with age and underlying medical conditions. The reported cost of influenza‐related hospitalizations was found to be up to 2.5 times higher among at‐risk populations compared with not‐at‐risk populations. This review documents the considerable economic impact of influenza among adults aged 18 to 64. In this age group, most of the influenza costs are indirect, which are generally not recognized by decision makers. Future studies should focus on at‐risk subgroups, lab‐confirmed cases, and European countries.

## 1 INTRODUCTION

Seasonal influenza outbreaks can occur every year worldwide, causing substantial morbidity and mortality. The World Health Organization estimates that worldwide each year, influenza epidemics result in up to 5 million severe cases of disease and up to 650,000 respiratory deaths across all ages.^1^ Due to population distribution in terms of absolute numbers, in any given year, most cases of influenza occur in adults aged 18 to 64. For example, in the United States during the 2017–2018 influenza season, there were 14.4 million influenza cases among 18‐ to 49‐year‐olds and 13.2 million cases among 50‐ to 64‐year‐olds compared with 5.9 million in the 65 and older age group and 11.2 million in children aged 18 or younger.^2^

Although influenza can affect any person and age group, certain populations are at greater risk of exposure to infection or of developing severe disease. Population groups at higher risk of developing severe outcomes are pregnant women, young children, adults aged 65 years and older, and those with underlying conditions.^3^ For example, in the United States, patients aged over 65 years account for 70% to 85% of influenza‐related deaths.^4^ In addition to the aforementioned at‐risk populations, healthcare workers (HCWs) are also a key population recommended for seasonal influenza vaccination. HCWs may be highly exposed to seasonal influenza viruses through contact with patients, which increases their own risk of illness and their potential to spread the disease to others.^5^

Influenza imposes a large economic burden to healthcare systems and to society. Notably, for the adult American population, a study published before the COVID‐19 pandemic has demonstrated that influenza accounted for 65% of the total economic burden caused by vaccine‐preventable diseases.^6^ The proportion of costs attributable to influenza was particularly high among the 19‐ to 49‐year‐old age group, in which influenza accounted for 85% of the vaccine‐preventable disease economic burden, compared with 67% for 50‐ to 64‐year‐olds and 55% among those aged 65 and older.^6^ Influenza costs originate from inpatient and outpatient care settings and substantial indirect costs related to lost productivity.^7^ In the United States, total annual direct medical costs have been estimated to be US$3.2 billion, whereas indirect costs accounted for US$8.0 billion. For the latter, 67% were engendered by the 18‐ to 64‐year‐old age group.^8^ In the European Union, costs of seasonal influenza are estimated at €6 billion to €14 billion annually.^9^

While the economic burden of influenza infection among adults aged 65 and older has been well described,^10^, ^11^, ^12^ less is known for other adult age groups. This systematic literature review (SLR) was conducted to describe the economic burden of seasonal influenza in adults aged 18 to 64 years, to identify the main determinants of direct and indirect costs, and to highlight any gaps in the existing published evidence. A stratification of the data by age group (18 to 49 years/50 to 64 years) and at‐risk status (at‐risk/general population) was completed to assess whether specific groups are associated with a higher economic burden.

## 2 METHODS

The SLR followed an a priori protocol that specified search terms, inclusion and exclusion criteria, and a methodological approach to the review aligned with the Preferred Reporting Items for Systematic Reviews and Meta‐Analyses (PRISMA) statement.^13^ The protocol was not publicly registered. Research questions and inclusion and exclusion criteria were defined in terms of population, intervention, comparator, outcomes, study design, and time frame (PICOS‐T), as seen in Table 1. The search of literature databases (MEDLINE via PubMed and Embase via Embase.com) was conducted on February 7, 2020. (Full search strategies are available in the Supporting Information.) Peer‐reviewed publications were searched from 2007 to the date of search and conference proceedings from 2018 to the date of search. In addition, bibliographies of relevant SLRs and meta‐analyses captured by the search were reviewed to identify any missing publications.

**TABLE 1:** PICOS‐T inclusion and exclusion criteria

Topic	Inclusion criteria	Exclusion criteria
Population	Patients aged 18–64 years infected with seasonal influenza virus, including laboratory‐confirmed cases and ILI^a^	Patients aged <18 or >65 years Patients infected during an influenza pandemic
Interventions/ comparators	Any/all/none	None
Outcomes	Direct costs: ○ Inpatient and outpatient costs ○ Costs of self‐management ○ Costs of complications arising from influenza virus infection Indirect costs ○ Costs of lost productivity for patients and caregivers ○ Costs of mortality attributed to influenza infection Amount of absenteeism/presenteeism for patients and caregivers Data were captured at both the aggregate level (e.g., total population costs) and the individual level (e.g., costs per healthcare contact per patient)	Non‐economic outcomes (e.g., epidemiology, efficacy, safety, and humanistic burden) Full economic evaluation data (e.g., cost‐effectiveness analyses results) without primary cost data
Study design	Real‐world observational studies (e.g., database studies, registries, prospective cohorts, surveys, and cross‐sectional studies) and randomized controlled and other trials that report cost data Cost‐effectiveness studies reporting primary cost data Any relevant SLRs and meta‐analyses were considered for hand‐searching of the reference lists	Case reports, case series (N ≤ 10) In vitro studies Animal studies Narrative reviews Editorials/opinion pieces
Timeframe	Publications from 2007 onward (for full‐text publications) Conference abstracts from 2018–2020	2006 or earlier (for full‐text publications) 2017 or earlier (for conference abstracts)
Geography	United States, Canada, Australia, United Kingdom, European Union 4 countries (i.e., Germany, France, Italy, Spain), and other high‐income countries in western Europe	Studies conducted in any other country
Language	English	Non‐English language publications

^a^ Specific subgroups of interest included at‐risk subgroups (defined as groups with a condition placing them at increased risk of influenza complications) including mainly pregnant women, patients with chronic underlying medical conditions, immunocompromised patients, and groups who can transmit influenza to these at‐risk subgroups.

Publications were screened against the inclusion and exclusion criteria shown in Table 1. First, the title and abstract of all unique references identified were screened to select publications for subsequent full‐text screening. Second, the full‐text versions of the selected publications were assessed for their suitability for inclusion. All publications were reviewed by two independent reviewers with resolution of any conflicts by a third independent reviewer. Only trials, real‐world observational studies, or cost‐effectiveness studies reporting at least one outcome of interest—primary direct or indirect cost or absenteeism data—for adults aged 18 to 64 were included. Full criteria are reported in Table 1.

Relevant data were fully extracted by one investigator and validated by a second independent investigator. Data elements extracted covered study characteristics, including method used to identify influenza cases, patient characteristics such as age group and presence of comorbidities, and cost and absenteeism data. If studies focused on cases who reported positive results of a laboratory test for influenza, they were categorized as lab‐confirmed influenza (LCI); if studies relied on the use of International Classification of Diseases (ICD)‐9/10 codes, presence of specified symptoms, READ codes, or patient surveys to identify influenza cases, they were classified as influenza‐like illness (ILI). The extracted cost and absenteeism data included total costs, direct costs, indirect costs, and proportion of patients missing work due to influenza and duration of absenteeism. When reported, the extracted direct costs items were hospitalization costs, emergency department (ED) costs, general practitioner (GP) costs, pharmacy costs, and overall direct costs; the indirect cost items extracted were costs of absenteeism, costs associated with premature death, and overall direct costs. Total costs referred to both direct and indirect costs combined, and overall direct and indirect costs referred to all subtypes of either direct or indirect costs, respectively, combined. Outcomes stratified by age group and risk profile were captured even when age group did not match those defined a priori (i.e., aged 18 to 49 and 50 to 64 years) and regardless of medical conditions implied.

## 3 RESULTS

**FIGURE 1:**
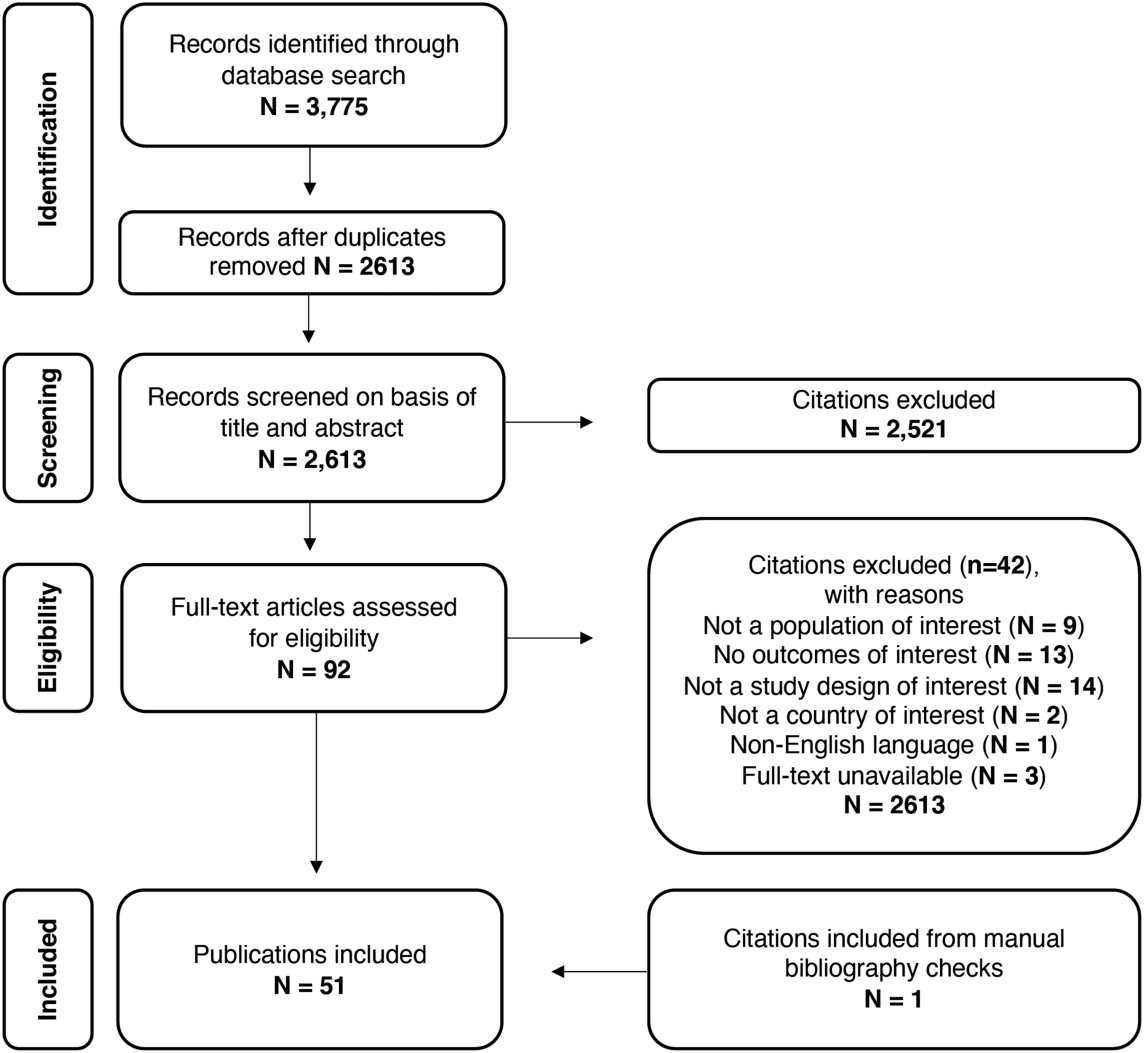
PRISMA diagram showing flow of literature

**FIGURE 2:**
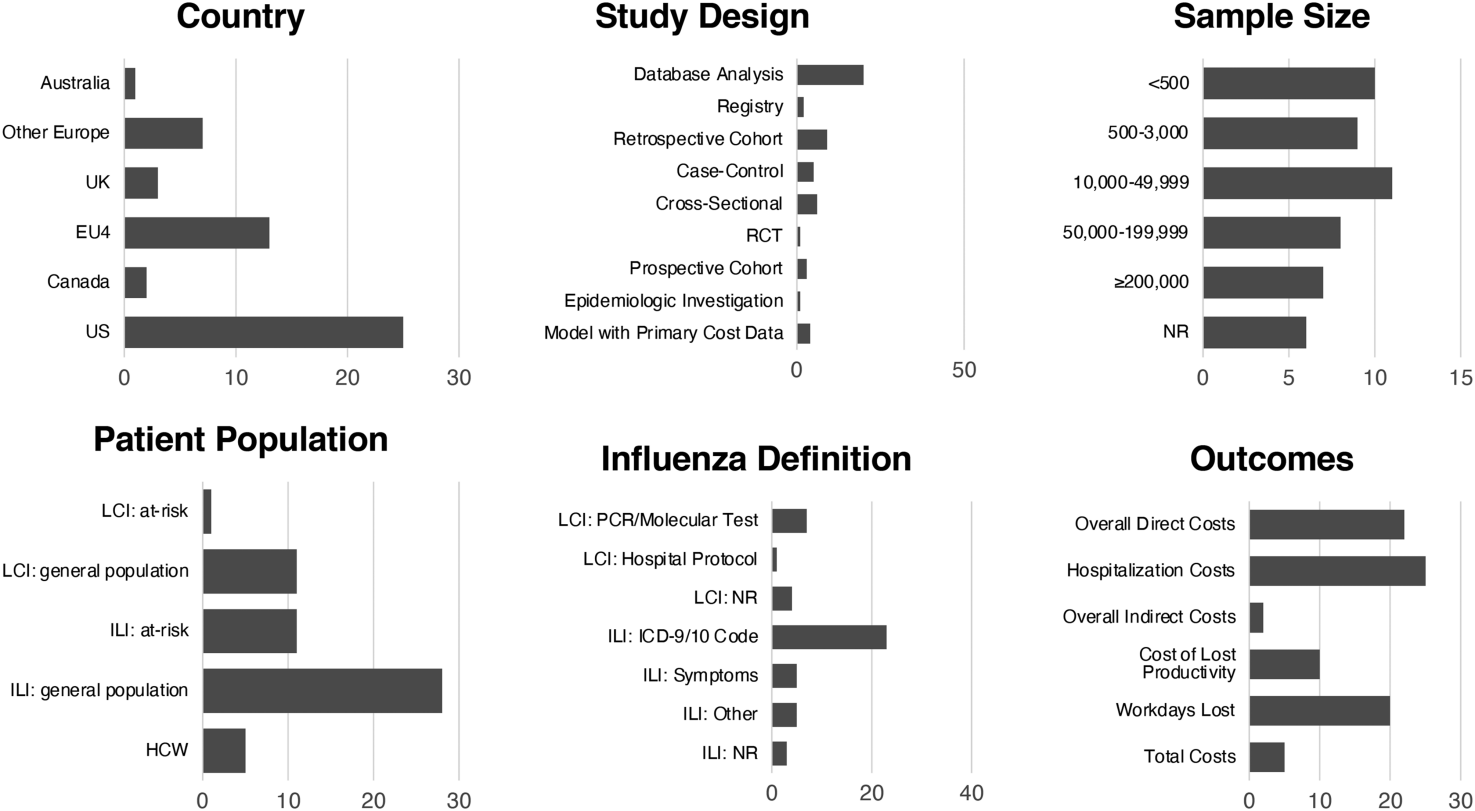
Characteristics of included studies. Abbreviations: EU4, European Union 4; HCW, healthcare worker; ICD, International Classification of Diseases; ILI, influenza‐like illness; LCI, laboratory‐confirmed influenza; NR, not reported; PCR, polymerase chain reaction; RCT, randomized controlled trial; UK, United Kingdom; US, United States

This SLR identified 51 studies reporting direct and indirect costs or workdays lost related to influenza in patients aged 18 to 64. See Figure 1 for the PRISMA diagram outlining full study attrition. Half of the included studies were conducted in the United States; 65% of studies used a retrospective design, often utilizing claims databases (Figure 2). Of studies reporting sample size, 58% reported data for samples of >10,000 individuals. While this review primarily sought to identify data stratified by the 18 to 49 and 50 to 64 age groups, the included studies utilized several different stratifications by age. For example, while most studies defined adults as ≥18, thresholds of 15, 16, 17, and 19 also were used; 18 to 49 and 50 to 64 were commonly reported age groups, but some studies subdivided patients into smaller age ranges, and some grouped all patients ≥59 or 60 together.

Identified studies included 12 reporting on LCI and 36 reporting on ILI. The other three studies examined the link between absenteeism and influenza season. In these latter studies, influenza was considered as an exposure factor such as a calendar period defined by epidemiological indicators.

Few studies reported data for at‐risk populations: 11 ILI studies and only one LCI study. Various terms were used in the literature to describe at‐risk populations without a clear or consistent definition. For consistency, this publication uses the term “at‐risk” to refer to studies that used the terms “at‐risk” or “high risk” or identified one of the at‐risk groups of interest as defined in the review methods (Table 1).

The most reported outcomes were hospitalization costs (25 studies), overall direct costs (22 studies), and workdays lost (20 studies). Costs of workplace absenteeism were reported in 10 studies, total costs in five studies, and overall indirect costs in two studies. All extracted cost data are available in the supplemental tables.

Finally, five studies looked at influenza costs specifically among HCWs and primarily focused on influenza‐related absenteeism.

### 3.1 Direct costs of influenza

#### 3.1.1 Overall direct costs

A total of 22 studies reported overall direct costs attributable to influenza, of which eight studies investigated costs in LCI and only two provided data for an at‐risk population among those looking at ILI. Thirteen studies reported individual costs (cost per influenza case), and 10 studies estimated aggregated costs at the population level across the specified age group.

Reporting of individual costs was heterogenous and included the following denominators: influenza case, influenza case medically attended, case hospitalized or presented to an ED, or hospitalized case. Moreover, some studies did not report all the direct costs items, and others did not specify how the costs were attributed to their categories.

On average, in Europe, overall direct costs ranged from €56 in Italy per self‐reported influenza case^14^ to €90 in Germany per medically attended case.^15^ In the United States, overall direct costs ranged from US$161 to US$363 per medically attended case.^16^, ^17^, ^18^ Higher overall direct costs were observed for patients visiting an ED or who were hospitalized.^16^, ^19^, ^20^, ^21^

Among patients aged 18 to 64 years, mean overall direct costs per influenza patient increased with age,^19^, ^22^, ^23^, ^24^ and the increase was still greater in the ≥65 population.^19^, ^22^, ^23^ Overall direct costs per case were higher among influenza patients with complications and more broadly among at‐risk patients. Indeed, a US study reported that the overall direct cost per medically attended case was 2.1 to 2.7 times higher in complicated cases compared to uncomplicated cases.^24^ Moreover, an Italian study showed that the overall direct cost per case who presented to an ED or who were hospitalized was 1.6 times higher among those >50 years old with at least one risk factor compared to those without risk factors.^19^

Concordantly with individual results, aggregated cost data showed that overall direct cost attributable to influenza increases with age and was significantly higher in the elderly compared to adults less than 65 years old.^25^, ^26^, ^27^ Finally, based on data reported in a German study, 50% of the overall direct costs of influenza are attributable to adults aged 18 to 59 years old.^23^

#### 3.1.2 Hospitalizations

Costs attributable to hospitalizations were a main driver of overall direct costs. US and Australian studies have shown that hospitalization costs accounted for 73 to 75% of overall direct costs of influenza in adult populations aged 15 or 18 to 64 years.^26^, ^27^

Twenty‐five studies reported hospitalization costs: 23 reported individual costs, and six reported aggregated costs. Only three studies reported hospitalization cost data for LCI cases across three different countries.^20^, ^22^, ^28^ Data on LCI patients were not stratified by age, precluding comparison. The format of the hospitalization cost data was quite heterogeneous from one study to another: some studies reported average cost per hospital stay while others reported hospitalization cost per influenza episode. Furthermore, some studies reported average costs across hospitalized patients only, while others included all cases regardless of hospitalization status. In most studies, hospitalization costs were extracted from claims databases using ICD codes associated with an influenza infection.

Cost per hospitalization was lower in Europe than in the United States. European cost per hospitalization ranged from €2033 among patients of all ages in Germany to €6827 among 50‐ to 64‐year‐olds in Belgium.^23^, ^29^ In the United States, cost per hospitalization ranged from US$7067 among all adult patients with uncomplicated influenza to US$38,662 among all 45‐ to 59‐year‐olds.^24^, ^30^

Across outcome definitions, individual hospitalization costs in the general population increased with age among patients under 65 years. Indeed, five studies reported higher hospitalization costs for those aged 50 to 64 (or 45 to 59) compared with those aged 18 to 49 (or 18 to 45).^24^, ^25^, ^26^, ^29^, ^30^ However, three studies reported lower individual costs among patients aged ≥60 or ≥65 years than for patients in the 45‐ to 59‐year‐old or 50‐ to 64‐year‐old age groups, respectively,^24^, ^25^, ^30^ while one reported a continued increase in individual costs for patients aged ≥65.^26^

Hospitalization costs were higher among complicated cases and at‐risk populations. Indeed, hospitalization costs were 1.4 to 1.5 and 1.1 to 2.0 times higher in complicated influenza cases than in uncomplicated cases among those 18 to 49 years old and 50 to 64 years old, respectively.^24^, ^29^ Moreover, five US studies and one Canadian study showed that cost of influenza‐attributable hospitalization is up to 2.5 times higher in an at‐risk population versus a not at‐risk population.^20^, ^25^, ^26^, ^31^, ^32^, ^33^ This increase seems to vary across conditions.^20^

Aggregated hospitalization costs by age were reported in two studies. These 2 studies reported higher hospitalization costs among those aged 50 to 64 compared with those aged 15 or 18 to 49.^26^, ^27^ Both studies highlighted that for elderly age groups, hospitalization costs are well above those seen in younger adults.

### 3.2 Indirect costs of influenza

#### 3.2.1 Overall indirect costs

Studies were categorized as overall indirect costs if they included combined data of multiple components of indirect costs. However, the specific indirect costs included in the evaluation of overall indirect costs varied between studies, making comparisons difficult.

Two studies reported overall indirect costs, both from the United States. Both studies reported aggregate costs only. Costs of absenteeism and lost productivity due to death were included in both studies,^25^, ^26^ and one additionally included costs of quality‐adjusted life‐years (QALYs) due to all‐cause influenza‐attributed mortality.^26^ One study reported on a representative national population^25^ while the other reported on the US Department of Veteran's Affairs (VA).^26^

Indirect costs were consistently higher for patients aged 50 to 64 than for patients aged 18 to 49.^25^, ^26^ Both studies showed that indirect costs are the main contributor to the economic burden of influenza, accounting for 83% and 99% of the total costs among the 18 to 64 years old, respectively, of the general population and veterans.^25^, ^26^

#### 3.2.2 Costs of influenza‐related absenteeism

As 18‐ to 64‐year‐olds comprise most of the working population, the burden of absenteeism due to influenza and its associated costs are of particular interest in this age group. Ten studies reported costs of influenza‐related absenteeism in 18‐ to 64‐year‐olds. Only one study provided data for LCI, one for an at‐risk population, and two for HCWs. Among these studies, nine reported individual costs, and four estimated aggregated costs.

Since some studies explored the average cost of absenteeism only among influenza patients using sick leave while others considered all influenza patients without specifying whether they had sick leave, individual absenteeism costs were classified into cost per case with sick leave and cost per case regardless of sick leave. Cost per influenza patient with sick leave ranged from €176 to €577 in Germany.^23^, ^34^ Cost per case regardless of sick leave was slightly lower, ranging from €93 in France to €424 in Germany.^14^, ^15^, ^17^, ^18^, ^22^

Overall, in the adult population, absenteeism costs per influenza case increased with age in the 18‐ to 64‐year‐old age group but decreased among those aged ≥65.^18^, ^23^ This decrease is likely explained by the lower employment rate among older patients. Moreover, a US study reported higher absenteeism costs in populations with underlying chronic conditions (diabetes, asthma, chronic cardiovascular, or lung disease) compared with the general population.^18^

For population aggregated costs, an Italian study showed that absenteeism costs are highest for those aged 40 to 59 years followed closely by those aged 50 to 59 years. As with individual costs, the lowest cost for absenteeism was seen for older individuals (aged ≥60).^35^ When limiting indirect costs to absenteeism costs, two studies reported that absenteeism costs account for 79% and 90% of the total economic burden of influenza in German adults aged 18 to 59 years old and all Norwegians, respectively.^23^, ^36^

#### 3.2.3 Workdays lost

Costs of lost working time are are directly driven by the amount of time lost due to absenteeism (time fully out of work) and presenteeism (time at work but not fully productive).

Twenty studies reported data for absenteeism associated with influenza among 18‐ to 64‐year‐olds. Of these, only two studies provided data for a population at‐risk, and four studies focused on HCWs. In addition, only two studies reported data for LCI cases. All studies reported individual data of absenteeism and one additionally reported aggregated data.

Identified studies generally reported two types of outcome: either the proportion of patients taking sick leave due to an influenza episode (7 studies) or the duration of absence from work (14 studies). Among studies reporting duration of absence, data were classified as average duration of workplace absence per influenza case with sick leave or average duration of workplace absence per influenza case regardless of taking sick leave (all patients).

For the proportion of influenza patients taking sick leave, most of the studies reported an average of 30% to 36%. For instance, in Germany, two studies estimated that 33.4% to 35.6% of influenza patients took sick leave.^23^, ^34^ In Belgium, an estimate of 34% was obtained among community influenza patients.^21^ Similar percentages were obtained in two US studies conducted over nine influenza seasons.^17^, ^18^ Some outliers were identified: a higher rate (59%) was found among adults aged 15 to 59 years in a French study conducted in patients infected with lab‐confirmed influenza B,^22^ and a German study showed that a sickness certificate was delivered for 60.7% of ILI episodes in adults, though no information was given on whether patients actually took this sick leave.^15^

The proportion of patients taking sick leave increased with age within the 18‐ to 64‐year‐old age group and for at‐risk populations compared with a general population in a US study.^18^ Moreover, as highlighted in a Belgian study, increased levels of healthcare resource utilization during an influenza episode are associated with more absenteeism. More patients presenting to an ambulatory clinic had an interruption of daily activity than did patients remaining in the community; all hospitalized patients experienced absenteeism.^21^

The reported average duration of workplace absence per influenza patient with sick leave ranged from 6.0 to 8.2 days in European studies^15^, ^22^, ^23^, ^34^, ^37^ and from 14 to approximately 24 working hours in studies from North America.^38^, ^39^, ^40^ In studies of influenza patients regardless of whether they had access to sick leave, reported durations of absenteeism were consistently lower, ranging from 0.6 days in the United States to 3.3 days in Germany.^15^, ^17^, ^18^, ^41^, ^42^

Across all populations of influenza patients considered, the average duration of absenteeism increased with age within the 18‐ to 64‐year‐old age group.^18^, ^23^, ^25^, ^37^, ^39^ Moreover, in two studies reporting workdays lost specifically for hospitalized patients, length of absence was substantially higher compared with outpatients.^21^, ^25^ Finally, data from US studies showed that the average duration of sick leave among the at‐risk population was up to 1.8 times higher compared with the general population.^18^, ^25^

Among the studies that focused on HCWs, three provided average lengths of influenza‐related sick leave. In Italy, where influenza is responsible for 11.9% of all‐cause work absences in HCWs, they had an average of 4.6 days of absence when they had influenza.^43^ Two additional European studies showed this duration varied substantially across categories of HCWs from less than 1 day to over a week.^44^, ^45^ Furthermore, a US study reported that each influenza season causes a median of 12 hours of working time lost per HCW aged ≥18 years, whether or not they contracted influenza.^46^

Only one study incorporated presenteeism as an outcome. While the length of workplace absence was generally lower in the United States compared with European countries, this study suggested that lost productivity is substantial. At 7 to 17 working days post‐disease onset, adults aged ≥18 years reported 67% of their working hours were lost due to absenteeism or presenteeism.^47^

### 3.3 Total costs of influenza

Total costs represent the complete economic burden of influenza. Total costs incorporate both direct and indirect costs, such as those due to absence from work or to premature death. However, studies were not always clear about the exact components included in the assessment of total costs.

Five studies reported the total costs of influenza. None of these studies reported data for at‐risk populations or HCWs and only one reported data for patients with LCI.

Three studies reported total costs on an individual basis (per case); these costs varied considerably between studies. The lowest costs were seen in France at €126.10 per case.^22^ However, this study was limited to lab‐confirmed cases of influenza B, while the other studies reported on ILI. In Italy, cost per episode was €38.71 from the perspective of the National Health Service and €140.33 from the family perspective.^14^ The highest costs were seen in Germany, with cost per episode ranging from €248 among adults ≥60 years to €584 for those aged 17 to 59.^15^ Two studies with age‐stratified data reported higher total costs for younger adults (15‐ to 64‐year‐olds or 17‐ to 59‐year‐olds) versus older adults (≥65 years or ≥60), largely driven by indirect costs.^14^, ^15^ The German study showed that 82% of the average total cost per episode is attributable to indirect cost in adults.^15^

Aggregated total costs increased with patient age within the 18 to 64 age group.^25^, ^26^ In the general US population, costs continued to increase for those aged ≥65,^25^ but in a population of US veterans, total economic burden was lower in those aged ≥65 than those 18 to 49 or 50 to 64.^26^ Both studies included costs due to absenteeism and premature death in indirect costs. Both studies enumerated direct costs for hospitalizations and outpatient visits, while the VA study also included ED costs. In the United States in 2007, total economic burden represented US$87,067.3 million, of which 10%, 21%, and 64% of costs were respectively attributable to 18‐ to 49‐year‐olds, 50‐ to 64‐year‐olds, and ≥65‐year‐olds.^25^

## 4 DISCUSSION

The economic burden of seasonal influenza is substantial among adults. Despite methodological heterogeneity observed across studies identified by this SLR, indirect costs have been reported to be the primary driver of total costs of influenza in adults, at both individual and population levels. Indeed, whatever the scope of indirect costs considered, they accounted for up to 82% of the total cost per influenza case^15^ and 70% to 95% of the total costs of influenza when measured at population level.^23^, ^25^, ^26^ Three studies provided data allowing to calculate this outcome by age group and showed that 79% and 83 to 99% of the economic burden of influenza is attributable to indirect costs respectively in populations aged 18 to 59 years and 18 to 64 years.^23^, ^25^, ^26^ These values are consistent with the 83% estimated in the 18‐ to 64‐year‐old population in a recent US study outside the scope of this review.^8^ When considering direct costs only, hospitalization costs were the main driver, with 73 to 75% of direct costs attributable to hospitalization.^26^, ^27^

Within the target age range of this review, older patients (i.e., those aged 50 to 64) generally had higher influenza‐associated costs compared with younger adults (i.e., those aged 18 to 49). Additionally, patients with one or more risk factors for influenza complications had higher costs than those patients not at risk. Healthcare resource utilization data were opportunistically extracted alongside costs and showed similar trends with age groups and the presence of risk factors. These data should reflect the fact that severe forms of influenza are more frequent with age and the presence of comorbidities.

While this review focused on adults aged 18 to 64 years, many of the included publications also reported data for elderly (≥65 years) patients, which were also opportunistically extracted. Across studies that provided age‐stratified data, the average total cost per case was higher in adults aged <65 than in the elderly. This could be related to the major role played by indirect costs, which are more substantial in those aged 18 to 64 partly due to higher employment rates. Conversely, studies have shown that the overall direct costs per case were higher in the elderly than in younger adults. This may be explained by the more severe impact influenza can have generally on these patients, which would result in increased healthcare use and therefore higher costs. Nevertheless, this trend was not observed when considering only hospitalization costs. Most of the studies reported higher hospitalization costs per case in patients aged 50 to 64 than in the elderly. One reason for this could be a kind of selection bias with the elderly more easily hospitalized due to the greater risk for complications in this age group. Hospitalized young adults may then have more severe disease than the elderly and require more medical intervention. It would be interesting to confirm this trend and hypothesis in future studies. However, when looking at hospitalization costs at population level, they were higher in elderly patients than in younger adults because of the increase of hospitalization incidence with age.^26^, ^27^

The ability to draw robust conclusions regarding the economic burden of influenza in adults is constrained by many limitations, first those inherent to systematic reviews and second those related to the included studies in this review.

While studies reporting costs due to influenza in patients aged 18 to 64 were identified systematically, no review can ensure that all available data for an outcome of interest have been identified. Notably, a recent study from 2017 looking at the impact of LCI on absence from work in England was not captured.^48^This study found that the percentage of patients aged 16 to 64 years old taking time off work for influenza ranged from 20% for influenza B to 31% for influenza A, values similar to those identified in this review.^48^ On another note, it might be surprising not to see the study from Putri et al^8^ among the identified publications. This is not an omission since this study was not based on primary data, which was an inclusion criterion, but on previously published works particularly that of Molinari et al^25^ that was identified in this review.

Regarding limitations related to the studies included, first, half of the included studies were conducted in the United States. As the US healthcare system functions very differently from European systems in relation to costs, results of these studies should not be generalized to other high‐income countries. This results in quite poor evidence on the economic burden of influenza in adults aged ≤65 outside the United States.

Second, only 11 studies reporting on patients with LCI were identified in this review. The others, which were categorized as ILI, relied on coding systems or symptoms and may have led to less specific results for influenza infection and may not fully reflect its true economic burden. Nevertheless, as ICD coding practices differ between countries, we classified a study as LCI only if the authors explicitly mentioned that the influenza cases were laboratory confirmed. The others were classified as ILI although in some studies, the authors focused on ICD codes that are theoretically specific to laboratory confirmed influenza. This choice may have excluded three studies from LCI category, as we ignore if ICD codes are appropriately informed.^15^, ^23^, ^34^

Third, only 12 studies measured the cost of influenza in at‐risk populations, and only one study provided data on at‐risk patients with LCI. No studies on at‐risk patients were available for total costs, overall direct costs, or overall indirect costs. Thus, literature on the economic burden of influenza is poor for the at‐risk population, particularly in Europe. This is an important evidence gap because most European countries mainly recommend influenza vaccination for individuals at risk of developing severe disease. Only five studies looked at HCWs, reporting primarily on absenteeism. This focus can be explained by the central role played by HCWs' sick leaves in health system disruption during the influenza season.

It is important to stress that results comparisons were made with caution due to high heterogeneity across studies and differences in definitions of the reported outcomes. For instance, age groups vary across studies: some reported cost data for people aged 16 to 59 years old, while others used the 18‐ to 64‐year‐old age group cutoff. Moreover, when risk groups were analyzed, they differed greatly from one study to another. At‐risk people referred either to people with a specific condition (e.g., patients with diabetes) or to a group of individuals with mixed chronic conditions generally undefined. Nonetheless, these last two limitations did not prevent this review from highlighting some trends in influenza costs.

For results reported at the individual level, the estimated value was in some studies an average among all influenza patients; in others, it was an average among patients who consumed this resource, and in others, the denominator was not specified. For instance, it was sometimes not clear if the estimated hospitalization cost was an average among all influenza patients or among hospitalized patients, and if the estimated duration of absenteeism was an average among all influenza patients or among patients with sick leave only.

Additionally, even if many studies reported the same composite costs such as overall direct or overall indirect costs, their scope varied from one study to another; some studies defined direct costs as the sum of hospitalizations and GP visits costs, while others added ED visits and pharmacy. Similarly, some studies restricted indirect costs to absenteeism‐related costs, whereas others also estimated costs associated with premature deaths. This required a careful interpretation of the data and in some cases reclassifying the extracted costs. In the same vein, there were difficulties in interpreting some studies which have estimated composite costs such as outpatient or inpatient costs without indicating their definition.

This review upholds the complexity of assessing the full economic burden of a disease.^49^ Even if measuring direct costs could be relatively straightforward when using insurance claims databases, estimating indirect costs is much trickier and is associated with significant uncertainty, particularly for absenteeism‐related data. Indeed, in most studies, absenteeism data were not collected from insurance databases or from employers' records, but from patient surveys or analyses of doctors' certificates to collect durations of sick leaves. Patient surveys are associated with at least response and recall biases, while doctors' certificates assess the prescribed duration of sick leave rather than the actual sick leave duration and underestimates absenteeism because only patients who visited a GP are counted. Given that indirect costs account for nearly 80% of the economic burden in adults aged 18 to 64 years,^8^ which represents most of the workforce, efforts should be made to better document influenza‐related absenteeism.

## 5 CONCLUSIONS

This review identified indirect costs as the main driver of economic burden due to influenza in the 18‐ to 64‐year‐old age group and hospitalizations as the main component of direct costs. Nevertheless, indirect costs are generally not recognized by decision makers, especially in economic evaluations, leading to an underestimation of the economic impact of influenza. Consistently across studies, influenza‐related costs increase with age and the presence of comorbidities within the 18‐ to 64‐year‐old age group.

This review highlighted substantial gaps and heterogeneity in the literature, limiting generalizability and interpretation.

Due to these gaps, there are multiple opportunities for future research. Areas of focus include economic burden on those at risk of severe outcomes, among patients with lab‐confirmed influenza, and on absenteeism due to influenza. Of note, evidence is missing on the economic burden across all European countries. Future investigations should be designed after reviewing the existing literature to attempt to standardize meaningful outcome and population definitions.

## AUTHOR CONTRIBUTIONS

**Caroline de Courville:** Conceptualization; data curation; funding acquisition; investigation; methodology; resources; validation; visualization; writing‐review and editing. **Sarah Cadarette:** Data curation; investigation; methodology; project administration; resources; validation; visualization; writing‐original draft. **Erika Wissinger:** Data curation; investigation; methodology; project administration; resources; supervision; validation; visualization; writing‐original draft. **Fabián Alvarez:** Conceptualization; funding acquisition; methodology; resources; supervision; validation; writing‐review and editing.

6

### PEER REVIEW

The peer review history for this article is available at https://publons.com/publon/10.1111/irv.12963.

## Supporting information




**Data S1.** Supporting Information
