# Supplementary material for: The economic burden of influenza among adults aged 18 to 64: A systematic literature review
Source: Influenza Other Respir Viruses. 2022 Feb 5;16(3):376–85. doi: 10.1111/irv.12963 (PMC8983919; doi:10.1111/irv.12963)
Supplement: Supplementary file 1 — Data S1. Supporting Information [file IRV-16-376-s001.docx]

# Supplemental Materials

## Search Strategies

Table S1. Search strategy for Embase (via Embase.com)

| **Sample Search** | | |
| --- | --- | --- |
| **Database** | **Embase (via Embase.com)** | |
| **Date of search** | **February 7, 2020** | |
| **Search** | **Query** | **Number of records found** |
| #1 | (influenza:ti OR flu:ti) NOT (child*:ti OR neonate*:ti OR juvenile*:ti OR infant*:ti OR adolescent*:ti OR pediatric*:ti OR paediatric*:ti OR school:ti) | 77,228 |
| #2 | cost:ti,ab OR costs:ti,ab OR costing:ti,ab OR fee:ti,ab OR fees:ti,ab OR budget:ti,ab OR budgets:ti,ab OR budgeting:ti,ab OR expenditure:ti,ab OR expenditures:ti,ab OR expense:ti,ab OR expenses:ti,ab OR spending:ti,ab OR spendings:ti,ab OR economic:ti,ab OR economics:ti,ab OR economical:ti,ab OR pharmacoeconomic:ti,ab OR pharmacoeconomics:ti,ab OR pharmacoeconomical:ti,ab | 1,061,531 |
| #3 | absentee:ti,ab OR absentees:ti,ab OR absenteeism:ti,ab OR presenteeism:ti,ab OR productivity:ti,ab OR 'work loss':ti,ab OR employment:ti,ab OR 'sick leave':ti,ab OR 'sick day':ti,ab OR 'sick days':ti,ab OR 'medical leave':ti,ab OR pvli:ti,ab OR pvle:ti,ab OR 'present value of lifetime income':ti,ab OR 'present value of lifetime earnings':ti,ab OR wage:ti,ab OR wages:ti,ab OR salary:ti,ab OR salaries:ti,ab OR income:ti,ab OR incomes:ti,ab OR earning:ti,ab OR earnings:ti,ab OR revenue*:ti,ab | 306,593 |
| #4 | 'resource use':ti,ab OR 'resource utilization':ti,ab OR 'resource utilisation':ti,ab OR 'healthcare use':ti,ab OR 'healthcare utilization':ti,ab OR 'healthcare utilisation':ti,ab OR 'health care use':ti,ab OR 'health care utilization':ti,ab OR 'health care utilisation':ti,ab OR 'resource consumption':ti,ab OR 'healthcare consumption':ti,ab OR 'health care consumption':ti,ab | 51,520 |
| #5 | #2 OR #3 OR #4 | 1,315,224 |
| #6 | #1 AND #5 | 3,800 |
| #7 | #6 NOT (‘animals’/exp NOT ‘humans’/exp) | 3,330 |
| #8 | #6 NOT (letter:it OR editorial:it)  Remove letters and editorials | 3,299 |
| #9 | #7 NOT ([review]/lim NOT (systematic OR (meta AND analy*)))  Remove narrative reviews | 2,934 |
| #10 | Limit: Publication Dates (2007-2020) | 2,333 |
| #11 | #5 NOT ([conference abstract]/lim AND [<1966-2017]/py)  Remove conference abstracts prior to 2018 | 1,953 |

Key: exp – explode; py – publication years; ti,ab – title, abstract.

Table S2. Search strategy for MEDLINE (via PubMed)

| **Sample Search** | | |
| --- | --- | --- |
| **Database** | **Medline (via PubMed)** | |
| **Date of search** | **February 7, 2020** | |
| **Search** | **Query** | **Number of records found** |
| #1 | (influenza[ti] OR flu[ti]) NOT (child*[ti] OR neonate*[ti] OR juvenile*[ti] OR infant*[ti] OR adolescent*[ti] OR pediatric*[ti] OR paediatric*[ti] OR school[ti]) | 67,270 |
| #2 | Cost[tiab] OR costs[tiab] OR costing[tiab] OR fee[tiab] OR fees[tiab] OR budget[tiab] OR budgets[tiab] OR budgeting[tiab] OR expenditure[tiab] OR expenditures[tiab] OR expense[tiab] OR expenses[tiab] OR spending[tiab] OR spendings[tiab] OR economic[tiab] OR economics[tiab] OR economical[tiab] OR pharmacoeconomic[tiab] OR pharmacoeconomics[tiab] OR pharmacoeconomical[tiab] | 821,905 |
| #3 | absentee[tiab] OR absentees[tiab] OR absenteeism[tiab] OR presenteeism[tiab] OR productivity[tiab] OR “work loss”[tiab] OR employment[tiab] OR “sick leave”[tiab] OR “sick day”[tiab] OR “sick days”[tiab] OR “medical leave”[tiab] OR PVLI[tiab] OR PVLE[tiab] OR “present value of lifetime income”[tiab] OR “present value of lifetime earnings”[tiab] OR wage[tiab] OR wages[tiab] OR salary[tiab] OR salaries[tiab] OR income[tiab] OR incomes[tiab] OR earning[tiab] OR earnings[tiab] OR revenue*[tiab] | 250,922 |
| #4 | “resource use”[tiab] OR “resource utilization”[tiab] OR “resource utilisation”[tiab] OR “healthcare use”[tiab] OR “healthcare utilization”[tiab] OR “healthcare utilisation”[tiab] OR “health care use”[tiab] OR “health care utilization”[tiab] OR “health care utilisation”[tiab] OR "resource consumption"[tiab] OR "healthcare consumption"[tiab] OR "health care consumption"[tiab] | 33,268 |
| #5 | #2 OR #3 OR #4 | 1,023,999 |
| #6 | #1 AND #5 | 3,175 |
| #7 | #6 NOT (animals[mh] NOT humans [mh]) | 2,744 |
| #8 | #7 NOT (case reports [pt] OR editorial [pt] OR letter [pt] OR comment [pt])  Remove letters, editorials, case reports, comments | 2,692 |
| #9 | #8 NOT (review[pt] NOT (systematic OR meta-analysis))  Remove narrative reviews | 2,358 |
| #10 | Filter: Publication Dates January 1, 2007 – December 31, 2020 | 1,822 |

Key: MH – major heading; pt – publication type; ti – title; tiab –title abstract.

## Supplemental Outcome Tables

Table S3 Total direct cost

| **Citation**  **Time period** | **Country** | **Risk status** | **Age group (years)** | **Total direct costs** | **Influenza definition** |
| --- | --- | --- | --- | --- | --- |
| **Individual cost** | | | | | |
| *Any influenza case* | | | | | |
| Silva 2014^22^  2010–2011 | France |  |  | **Mean (SD) cost per influenza B case** | LCI B cases |
|  |  | General population | 0–84 | € 50.6 (184.7) |  |
|  |  |  | 15–64 | € 33.4 (19.8) |  |
|  |  |  | ≥65 | € 41.8 (18.2) |  |
| Ehlken 2015^15^  2010–2012 | Germany | General population | ≥17 | **Total direct cost per episode: € 90 with the following split:**  Payer perspective (physician visits, other physicians, hospital admissions, drugs): € 59  Patient perspective (copayment, transportation): € 15  Not covered by payer (physician visits): € 16 | ILI |
| Scholz 2019^23^  2012–2014 | Germany |  |  | **Total direct costs per average influenza case (including inpatient influenza + complications, outpatient, pharmaceutical)** | ILI |
|  |  | General population | 18–34 | € 67.85 |  |
|  |  |  | 35–59 | € 74.25 |  |
|  |  |  | ≥60 | € 131.59 |  |
| Dal Negro 2018^14^  March 2017 | Italy | General population | Age NR | **Total direct cost per episode of influenza or influenza-like syndrome**  NHS: € 38.71±8.39; family perspective: € 16.81 including:  **Vaccination** NHS: € 2.46±0.14, family perspective: € 1.81±0.12  **Resource consumption** NHS: € 27.81±7.92, family perspective: NA  **Drugs** NHS: € 8.44±0.40, family perspective: € 15.01±0.60 | ILI |
| Karve 2013a^17^  2002–2009 | US | General population | Age NR | **Total influenza-related medical cost per episode, mean**  Matched season: US$ 239.44  Mismatched season: US$ 300.83 | ILI |
| Karve 2013c^18^  2008–2009 | US | General population | All ages | **Mean total direct influenza-related healthcare costs per patient among influenza cases**  US$ 254.14–US$ 363.41 | ILI |
| Klesper 2015^16^  2011–2013 | US | General population | ≥19 | **Mean cost per episode** (unclear if among influenza cases or all patients)  US$ 160.54 (median US$ 105.64) | ILI |
| *Medically attended influenza cases* | | | | | |
| Berthod 2015^50^  2008–2012 | Switzerland | General population | ≥18 | Patients managed with iRDT: € 581, without iRDT: € 661  Probability of influenza ≥70%: € 493 (400–586)  Probability of influenza 40%–≤69%: € 615 (469–761)  Probability of influenza <40%: € 668 (451–885) | LCI |
| Karve 2013a^17^  2002–2009 | US | General population | Age NR | **Total influenza-related medical costs among patients with ≥1 influenza-related encounter (including pharmacy), mean**  Matched season: US$ 239.43  Mismatched season: US$ 300.43 | ILI |
| Karve 2013b^24^  1998–2009 | US |  |  | **Risk-adjusted predicted cost per patient with at least 1 medical encounter over 12 months (ED, GP, lab, HH/DME, pharmacy, ancillary care, hospital outpatient, inpatient)** | ILI |
|  |  | General population | ≥18 | Complicated case: US$ 3,422  Uncomplicated case: US$ 1,510 |  |
|  |  |  | 18–49 | Complicated case: US$ 2,747  Uncomplicated case: US$ 5,004 |  |
|  |  |  | 50–64 | Complicated case: US$ 5,004  Uncomplicated case: US$ 1,963 |  |
|  |  |  | ≥65 | Complicated case: US$ 5,766  Uncomplicated case: US$ 2,143 |  |
| Klesper 2015^16^  2011–2013 | US | General population | ≥19 | **30-day influenza-related medical cost based on initial place of service**  ED: US$ 423.04  Inpatient: US$ 9,924.07  Ambulatory/outpatient: US$ 139.17 | ILI |
| Bilcke 2014^21^  January–March 2012 | Belgium | General population | All ages | **Total direct costs per ILI episode (drug + consultation, no hospitalization costs)**  Community patients: € 3–€ 7  Ambulatory patient ILI: € 51–€ 64  Ambulatory patient, likely influenza (more specific than ILI): € 53–€ 67  Hospitalized patient, ILI: € 120–€ 140  Hospitalized patient, likely influenza: € 72–€ 83  Total cost = cost of NHS + patients + insurers, and likely flu more specific than ILI | ILI |
| *ED and hospitalized influenza cases* | | | | | |
| Trucchi 2019^19^  2011–2017 | Italy |  |  | **Mean direct costs of ILI/LRTI ED accesses and hospitalizations, government payer perspective** | ILI |
|  |  | General population | ≥50 | € 3,257 |  |
|  |  |  | 50–54 | € 1,837 |  |
|  |  |  | 55–59 | € 2,170 |  |
|  |  |  | 60–64 | € 2,861 |  |
|  |  | No risk factors | ≥50 | € 2,534 |  |
|  |  |  | 50–54 | € 1,459 |  |
|  |  |  | 55–59 | € 1,647 |  |
|  |  |  | 60–64 | € 2,037 |  |
|  |  | ≥1 risk factor | ≥50 | € 3,440 |  |
|  |  |  | 50–54 | € 2,232 |  |
|  |  |  | 55–59 | € 2,652 |  |
|  |  |  | 60–64 | € 3,230 |  |
| *Hospitalized cases* | | | | | |
| Ng 2018^20^  2010–2013 | Canada | General population | ≥16 | **Total cost per hospitalized case (GP, ED, hospitalization, medical, readmission within 30 days; 61.7% complications in hospital)**  All patients: CA$ 14,612  Alive: CA$ 13,929  Dead: CA$ 21,293  ICU stay: CA$ 39,477  No ICU stay: CA$ 10,427 | LCI |
| Soto 2016^51^  2013–2014 | Spain | General population | ≥18 | **Total cost per hospitalized patient** (reagents, technical staff, antivirals, disposables used by HCP and those due to family visits, extra nursing time due to isolation compared to non-isolated)  PCR: € 458.8, PCR/Xpert: € 463.90 | LCI |
| **Aggregated costs** | | | | | |
| Davis 2017^52^  2014–2015 | UK | General population | All ages | **Total cost** (n=1,000)  With isolation resource constraints  PCR strategy: £133,850  Alere iNPT: £43,190  Without isolation resource constraints  PCR strategy: £350,000  Alere iNPT: £261,590 | LCI |
| Brachman 2019^53^  NR | Germany | General population | Age NR | **Economic effect of implementing rapid test diagnostic vs PCR for influenza season**  € 31,892 across influenza season | LCI |
| Lankelma 2019^54^  2016–2018 | The Netherlands | General population | Age NR | **Savings related to implementation of PCR-based POCT for influenza on ED wait time:** € 397,030  (n=813 patients positive for influenza) | LCI |
| Hassoun 2017^55^  2013–2014 | US | General population | Age NR | **Savings of target enriched multiplex PCR respiratory panel vs rapid antigen influenza diagnostic test:** US$ 208,981.65 (n=246 patients with ILI) | LCI |
| Molinari 2007^25^  2001–2003 | US |  |  | **Total annual medical cost** | ILI |
|  |  | General population | All ages | US$ 10.4 billion (n=24,668,939) |  |
|  |  |  | 18–49 | US$ 1.83 billion (n=8,903,764) |  |
|  |  |  | 50–64 | US$ 2.76 billion (n=3,098,284) |  |
|  |  |  | ≥65 | US$ 4.15 billion (n=3,232,826) |  |
| Young-Xu 2017^26^  2010–2014 | US |  |  | **Total annual direct cost** | ILI |
|  |  | General population (VA patients) | ≥18: | US$ 48 million (n=5,540,636) |  |
|  |  |  | 18–49 | US$ 2,100,000 (n=1,168,625) |  |
|  |  |  | 50–64 | US$ 7,500,000 (n=1,517,931) |  |
|  |  |  | ≥65 | US$ 38,000,000 (n=2,854,080) |  |
| Newall 2008^27^  2000–2006 | Australia |  |  | **Total GP and hospitalization costs** | ILI |
|  |  | General population | All ages | US$ 114,885,308 (n=NR) |  |
|  |  |  | 15–49 | US$ 15,130,210 (n=NR) |  |
|  |  |  | 50–64 | US$ 15,563,946 (n=NR) |  |
|  |  |  | 65–74 | US$ 23,917,692 (n=NR) |  |
|  |  |  | 75–84 | US$ 54,730,712 (n=NR) |  |
| Scholz 2019^23^  2012–2014 | Germany | General population | All ages | **Total costs per year**  € 78,278,429 (range: 52,879,376–120,537,590; n=23,702 [2012]; n=48,786 [2013]; n=22,601 [2014]) | ILI |
| Xue 2010^36^  2005–2006 | Norway | General population | Age NR | **Mean direct costs of seasonal influenza for 1 year**  US$ 21,647,339 USD (n=4,640,219) | ILI |
| Sendi 2020^45^  2017 | Switzerland | General population | Age NR | **Economic burden at the rehabilitation center level** (n=26)  Total direct medical cost: US$ 9,974  Total direct cost: US$ 26,837 | ILI |

Key: CA$ -- Canadian dollar; DME – durable medical equipment; ED – emergency department; GP – general practitioner; HCP – healthcare provider; HH – home health; ICU – intensive care unit; ILI – influenza-like illness; iNPT – influenza near patient test; iRDT – influenza rapid diagnostic test; LCI – laboratory-confirmed influenza; LRTI – lower respiratory tract infection; NA – not applicable; NHS – National Health Service; NR – not reported; PCR – polymerase chain reaction; POCT – point of care test; SD – standard deviation; UK – United Kingdom; US – United States; US$ – United States dollar; VA – Veteran’s Affairs.

Table S4. Hospitalization cost

| **Citation**  **Time period** | **Country** | **Risk status** | **Age group (years)** | **Hospitalization costs** | **Influenza definition** |
| --- | --- | --- | --- | --- | --- |
| **Individual cost** | | | | | |
| *Hospitalized case* | | | | | |
| Ghazi 2016^28^  2012–2014 | US | General population | ≥18 | **Total hospitalization costs per hospitalized patient, median**  US$ 7,553 | LCI |
| Silva 2014^22^  2010–2011 | France |  |  | **Mean hospital cost among hospitalized influenza B patients** | LCI |
|  |  | General population | 0–84 | € 17.9 (n=2) |  |
|  |  |  | 15–64 | No hospitalized patients |  |
| Ng 2018^20^  2010–2013 | Canada |  |  | **Mean cost per LCI case requiring hospitalization** | LCI |
|  |  | General population | ≥16 | ICU stay: CA$ 3,772  General ward: CA$ 7,720  Total during admission cost (lab tests, VRE screens, procedures, medications): CA$ 14,031 |  |
|  |  | All at risk | ≥16 | CA$ 14,612 |  |
|  |  | Cardiac comorbidity | ≥16 | CA$  15,206 |  |
|  |  | Renal comorbidity | ≥16 | CA$  17,676 |  |
|  |  | COPD | ≥16 | CA$  15,928 |  |
| Aggarwal 2018^30^  2015 | US |  |  | **Total hospital cost per admissions** | ILI |
|  |  | General population | All ages | US$ 35,248 |  |
|  |  |  | 18–45 | US$ 32,978 |  |
|  |  |  | 45–59 | US$ 38,662 |  |
|  |  |  | ≥60 | US$ 34,935 |  |
| Karve 2013a^17^  2002–2009 | US | General population | Age NR | **Total influenza-related outpatient costs among patients with  ≥1 outpatient visit:**  Matched season: US$ 8,208.03  Mismatched season: US$ 9,555.66 | ILI |
| Karve 2013b^24^  1998–2009 | US |  |  | **12-month risk-adjusted mean hospitalization cost per patient receiving that service** | ILI |
|  |  | General population | ≥18 | Inpatient complicated influenza: US$ 10,790  Inpatient uncomplicated influenza: US$ 7,067  Outpatient complicated influenza: US$ 1,424  Outpatient uncomplicated influenza: US$ 981 |  |
|  |  |  | 18–49 | Inpatient complicated influenza: US$ 9,497  Inpatient uncomplicated influenza: US$ 7,023  Outpatient complicated influenza: US$ 1,299  Outpatient uncomplicated influenza: US$ 911 |  |
|  |  |  | 50–64 | Inpatient complicated influenza: US$ 14,530  Inpatient uncomplicated influenza: US$ 7,316  Outpatient complicated influenza: US$ 1,809  Outpatient uncomplicated influenza: US$ 1,149 |  |
|  |  |  | ≥65 | Inpatient complicated influenza: US$ 8,149  Inpatient uncomplicated influenza: US$ 2139  Outpatient complicated influenza: US$ 1,218  Outpatient uncomplicated influenza: US$ 1,293 |  |
| Karve 2013c^18^  2008–2009 | US | General population | All ages | **Total influenza-related inpatient costs among patients with  ≥1 influenza-related inpatient stay**  US$ 15,842.26–US$ 8,193.62  **Total influenza-related outpatient costs among patients with  ≥1 influenza-related outpatient encounter**  US$ 212.67–US$ 225.79 | ILI |
| Yandrapalli 2018^56^  2014 | US | General population | ≥18 | **Cost of care**  Mean: US$ 10,729; median: US$ 6,082 | ILI |
| Kiser 2018a^57^  2010–2014 | US | General population | ≥18 | **Median hospital cost per ICU patient treated with at least 2 days of oseltamivir**  Standard-duration oseltamivir group: US$ 59,433  Extended-duration oseltamivir group: US$ 68,085 | ILI |
| Kiser 2018b^58^  2010–2014 | US | General population | ≥18 | **Median hospital cost per ICU patient treated with at least 2 days of oseltamivir**  High oseltamivir dosage group: US$ 71,594  Standard oseltamivir dosage group: US$ 54,225 | ILI |
| Scholz 2019^23^  2012–2014 | Germany | General population | All ages | **Average inpatient cost per influenza (principal diagnosis) hospitalization**  € 2,033 | ILI |
| Crott 2014^29^  2002–2007 | Belgium |  |  | **Total hospitalization cost for flu and pneumonia per hospitalized patient** | ILI |
|  |  | General population | All ages | All: € 5,779; with complications: € 7,159 |  |
|  |  |  | 18–49 | All: € 4,493; with complications: € 6,777 |  |
|  |  |  | 50–64 | All: € 6,827; with complications: € 7,607 |  |
| Ghanbar 2019^32^  2016 | US |  |  | **Hospital cost related to influenza per inpatient** | ILI |
|  |  | Atrial fibrillation | Age NR | US$ 48,888 |  |
|  |  | No atrial fibrillation | Age NR | US$ 35,906 |  |
| Panhwar 2019b^31^  2013–2014 | US |  |  | **Mean hospital cost per hospitalization for influenza** | ILI |
|  |  | HF | ≥18 | US$ 11,609 |  |
|  |  | No HF | ≥18 | US$ 9,003 |  |
| Venkatesh 2018^33^  2000–2012 | US |  |  | **Hospitalization cost per discharge with influenza and RA as diagnoses** | ILI |
|  |  | RA | All ages | US$ 15,826 |  |
|  |  | No RA | All ages | US$ 5,953 |  |
| Cardoso 2019^59^  2010–2014 | US |  |  | **Median hospital cost per discharge with AMI diagnosis** | ILI |
|  |  | AMI with influenza | ≥18 | US$ 15,856 |  |
|  |  | AMI without influenza | ≥18 | US$ 15,339 |  |
| Panhwar 2019a^60^  2013–2014 | US |  |  | **Mean hospital cost per hospitalization for HF** | ILI |
|  |  | HF with influenza | ≥18 | US$ 12,137 |  |
|  |  | HF without influenza | ≥18 | US$ 12,003 |  |
| Skinner 2018^61^  2013–2018 | US |  |  | **Total secondary care costs for influenza vs non-influenza patients** | ILI |
|  |  | Chronic respiratory disease | ≥18 | £28,078 vs £18,005 (*P*<0.001) |  |
|  |  | Chronic heart disease | ≥18 | £33,242 vs £20,520 (*P*<0.0001) |  |
|  |  | Diabetes | ≥18 | £31,375 vs £20.142 |  |
| *Any influenza case* | | | | | |
| Karve 2013a^17^  2002–2009 | US | General population | Age NR | **Total influenza-related inpatients costs per total influenza cases, mean**  Matched season: US$ 124.61  Mismatched season: US$ 167.01 | ILI |
| Ehlken 2015^15^  2010–2012 | Germany | General population | ≥17 | **Cost of hospital admissions with relevant diagnoses per physician attended influenza**  Mean (SD): € 14 (178)  Median (range): € 0 (0–6,002) | ILI |
| Dal Negro 2018^14^  March 2017 | Italy | General population | Age NR | **Mean annual hospitalization cost** (denominator unclear, appears to be all ILI)  NHS perspective: € 24.43 (10.89)  Family perspective: NA | ILI |
| Newall 2008^27^  2000–2006 | Australia | General population | All ages | **Age-adjusted mean cost for influenza-attributable hospitalization per patient with health claim for influenza/ILI**  AU$ 5,413.20 | ILI |
| *Other* | | | | | |
| Young-Xu 2017^26^  2010–2014 | US |  |  | **5-season average cost per hospitalization per patient with at least  1 healthcare encounter the previous season** | ILI |
|  |  | Low risk (VA patients) | 18–49 | AU$10,872 |  |
|  |  |  | 50–64 | AU$13,705 |  |
|  |  |  | ≥65 | AU$14,723 |  |
|  |  | High risk (VA patients) | 18–49 | AU$13,673 |  |
|  |  |  | 50–64 | AU$14,623 |  |
|  |  |  | ≥65 | AU$14,839 |  |
| Molinari 2007^25^  2001–2003 | US |  |  | **Mean cost per hospitalization** | ILI |
|  |  | General population | 18–49 | US$ 19,012 |  |
|  |  |  | 50–64 | US$ 22,304 |  |
|  |  |  | ≥65 | US$ 11,451 |  |
|  |  | High risk | 18–49 | US$ 47,722 |  |
|  |  |  | 50–64 | US$ 41,309 |  |
|  |  |  | ≥65 | US$ 16,750 |  |
| **Aggregated costs** | | | | | |
| Molinari 2007^25^  2001–2003 | US | General population | All ages | **Total annual cost for hospitalization**  US$ 6.0 billion | ILI |
| Yandrapalli 2018^56^  2014 | US | General population | ≥18 | **Total costs of patients hospitalized for influenza and survived to discharge** (n=46,117)  US$ 489.9 million | ILI |
| Newall 2008^27^  2000–2006 | Australia | General population |  | **Hospitalization costs for claims for ICD influenza/pneumonia** | ILI |
|  |  |  | All ages | AU$ 44,482,794 |  |
|  |  |  | 0–4 | AU$ 3,398,468 |  |
|  |  |  | 5–14 | AU$ 798,831 |  |
|  |  |  | 15–49 | UA$ 6,142,525 |  |
|  |  |  | 50–64 | AU$ 5,573,748 |  |
|  |  |  | 65–74 | AU$ 7,133,248 |  |
|  |  |  | 75–84 | AU$ 22,940,768 |  |
|  |  |  | ≥85 | AU$ 10,005,189 |  |
|  |  |  |  | **Hospitalization costs for other respiratory illness attributable to influenza (ICD J, excluding J10-18)** |  |
|  |  |  | All ages | AU$ 60,052,914 |  |
|  |  |  | 0–4 | NS |  |
|  |  |  | 5–14 | NS |  |
|  |  |  | 15–49 | AU$ 3,104,973 |  |
|  |  |  | 50–64 | AU$ 7,691,553 |  |
|  |  |  | 65–74 | AU$ 16,310,432 |  |
|  |  |  | 75–84 | AU$ 22,940,768 |  |
|  |  |  | ≥85 | AU$ 10,005,189 |  |
| Xue 2010^36^  2005–2006 | Norway | General population | All ages | **Mean (95% CI) inpatient cost of seasonal influenza**  US$ 14,687,272 (US$ 6,959,764, US$ 27,161,845) | ILI |
| Young-Xu 2017^26^  2010–2014 | US |  |  | **Hospitalization only annual average VA cost** | ILI |
|  |  | Low risk (VA patients) | ≥18 | US$ 36,143,000 (n=5,540,636) |  |
|  |  |  | 18–49 | US$ 1,712,000 (n=1,168,625) |  |
|  |  |  | 50–64 | US$ 5,465,000 (n=1,517,931) |  |
|  |  |  | ≥65 | US$ 28,966,000 (n=1,445,967) |  |
|  |  | High risk (VA patients) | ≥18 | US$ 36,036,000 (n=2,089,948) |  |
|  |  |  | 18–49 | US$ 1,702,000 (n=128,037) |  |
|  |  |  | 50–64 | US$ 5,425,000 (n=553,798) |  |
|  |  |  | ≥65 | US$ 28,909,000 (n=1,408,113) |  |
| Pockett 2015^62^  2001–2009 | UK |  |  | **Total cost of inpatient admissions for ILI patients with at least  1 GP consultation for ILI** | ILI |
|  |  | Low risk, vaccinated | Age NR | All  Database sample: £3,524,668  UK population: £127,836,164  With complications  Database sample: £3,524,668  UK population: £127,836,164  Without complications  No data |  |
|  |  | Low risk, unvaccinated | Age NR | All  Database sample: £3,017,028  UK population: £109,424,572  With complications  Database sample: £2,997,820  UK population: £108,727,917  Without complications  Database sample: £19,208  UK population: £696,655 |  |
|  |  | High risk, vaccinated | Age NR | All  Database sample: £5,216,344  UK population: £189,191,551  With complications  Database sample: £5,216,344  UK population: £189,191,551  Without complications  No data |  |
|  |  | High risk, unvaccinated | Age NR | All  Database sample: £2,043,594  UK population: £74,119,099  With complications  Database sample: £2,035,362  UK population: £73,820,533  Without complications  Database sample: £8,232  UK population: £298,566 |  |

**Key:** AMI – acute myocardial infarction; AU$ -- Australian dollar; CA$ -- Canadian dollar; CI – confidence interval; COPD – chronic obstructive pulmonary disease; GP – general practitioner; HF – heart failure; ICD – International Classification of Diseases; ICU – intensive care unit; ILI – influenza-like illness; LCI – laboratory-confirmed influenza; NA – not applicable; NHS – National Health Service; NR – not reported; RA – rheumatoid arthritis; SD – standard deviation; UK – United Kingdom; US – United States; US$ -- US dollar; VA – Veteran’s Affairs; VRE – vancomycin-resistant *Enterococci*.

Table S5. Total indirect costs

| **Citation**  **Time period** | **Country** | **Risk status** | **Age group (years)** | **Total indirect costs** | **Influenza definition** |
| --- | --- | --- | --- | --- | --- |
| Molinari 2007^25^  2001–2003 | US |  |  | **Total annual lost earning due to absence and due to death** | ILI |
|  |  | General population | 18–49 | US$ 2,546.6 million |  |
|  |  |  | 50–64 | US$ 3,021.1 million |  |
|  |  |  | ≥65 | US$ 8,854 million |  |
| Young-Xu 2017^26^  2010–2014 | US |  |  | **Total annual cost of lost productivity of seasonal influenza** | ILI |
|  |  | General population (VA patients) | ≥18 | US$ 27 billion |  |
|  |  |  | 18–49 | US$ 1.3 million |  |
|  |  |  | 50–64 | US$ 12 million |  |
|  |  |  | ≥65 | US$ 14 million |  |
|  |  |  |  | **Annual monetary value of QALYs lost caused by influenza-attributed all-cause deaths averaged over 5 respiratory seasons** |  |
|  |  | General population | ≥18 | US$ 1,103 million |  |
|  |  |  | 18–49 | US$ 305 million |  |
|  |  |  | 50–64 | US$ 791 million |  |
|  |  |  | ≥65 | US$ 6.2 million |  |
|  |  | High risk | ≥18 | US$ 1,078 million |  |
|  |  |  | 18–49 | US$ 301 million |  |
|  |  |  | 50–64 | US$ 771 million |  |
|  |  |  | ≥65 | US$ 5.7 million |  |
|  |  | Low risk | ≥18: | US$ 25 million |  |
|  |  |  | 18–49 | US$ 3.8 million |  |
|  |  |  | 50–64 | US$ 20 million |  |
|  |  |  | ≥65 | US$ 0.5 million |  |
|  |  |  |  | **Annual estimates of lost productivity for influenza-attributed healthcare encounters and all-cause mortality over 5 respiratory seasons** |  |
|  |  | General population | ≥18 | ED visits: US$ 590,000  Hospitalization only: US$ 626,000  Hospitalization with extended care: US$ 197,000  All-cause mortality: US$ 25,758,000  Total: US$ 27 million |  |
|  |  |  | 18–49 | ED visits: US$ 25,000  Hospitalization only: US$ 69,100  Hospitalization with extended care: US$ 10,000  All-cause mortality: US$ 1,221,000  Total: US$ 1.3 million |  |
|  |  |  | 50–64 | ED visits: US$ 355,000  Hospitalization only: US$ 207,000  Hospitalization with extended care: US$ 82,000  All-cause mortality: US$ 11,049,000  Total: US$ 12 million |  |
|  |  |  | ≥65 | ED visits: US$ 229,000  Hospitalization only: US$ 350,000  Hospitalization with extended care: US$ 105,000  All-cause mortality: US$ 13,488,000  Total: US$ 14 million |  |

Key: ED – emergency department; ILI – influenza-like illness; QALY – quality-adjusted life-year; US – United States; US$ -- US dollar; VA – Veterans Affairs.

Table S6. Cost of influenza-related absenteeism

| **Citation**  **Time period** | **Country** | **Risk status** | **Age group (years)** | **Cost of influenza-related absenteeism** | **Influenza definition** |
| --- | --- | --- | --- | --- | --- |
| **Individual cost** | | | | | |
| *Cost of workplace absenteeism per influenza case regardless of sick leave* | | | | | |
| Silva 2014^22^  2010–2011 | US | General population | All ages | **Daily allowances per influenza B case:** € 92.7 (SD: 174.2) | LCI |
| Karve 2013a^17^  2002–2009 | US | General population | Age NR | Matched season: US$ 175.1; unmatched season: US$ 237.3  Average US$ 209.66 | ILI |
| Ehlken 2015^15^  2010–2012 | Germany | General population | ≥17 | Mean: € 424; median: € 385 | ILI |
| Dal Negro 2018^14^  March 2017 | Italy | General population | Age NR | **Cost from Italian family perspective:** € 123.52 | ILI |
| Karve 2013c^18^  2008–2009 | US |  |  | **Total costs associated with influenza-related workplace absence, per influenza case** | ILI |
|  |  | General population | All ages | US$ 226.34–US$ 279.50 |  |
|  |  |  | 18–24 | US$ 100.88–US$ 146.56 |  |
|  |  |  | 25–44 | US$ 187.95–US$ 233.16 |  |
|  |  |  | 45–64 | US$ 269.13–US$ 376.85 |  |
|  |  | Asthma | All ages | US$ 247.98–US$ 489.36 |  |
|  |  | Diabetes | All ages | US$ 222.85–US$ 386.20 |  |
|  |  | CV disease | All ages | US$ 267.14–US$ 604.38 |  |
|  |  | Chronic lung disease | All ages | US$ 256.86–US$ 460.16 |  |
| *Cost of workplace absenteeism per influenza case with sick leave* | | | | | |
| Scholz 2019^23^  2012–2014 | Germany |  |  | **Costs for working days lost per case** | ILI |
|  |  | General population | 18–34 | € 370.80 |  |
|  |  |  | 35–59 | € 673.84 |  |
|  |  |  | ≥60 | € 569.46 |  |
| Haas 2014^34^  2012–2013 | Germany | General population | Age NR | **Cost per incapacity to work per day:** € 176 | ILI |
| Sendi 2020^45^  2017 | Switzerland | HCW | Age NR | **Cost per HCW who had work absence** (n=33)  CHF 2,636.4  € 2,463.9  USD 2,584.7 | ILI |
| **Aggregated data** | | | | | |
| *Total annual cost of workdays lost due to influenza* | | | | | |
| Xue 2010^36^  2005–2006 | Norway | General population | Age NR | **Annual indirect cost of influenza (seems to consider only sick leave):**  Employees’ own sickness: US$ 202,247,136  Employees’ children’s sickness: US$ 28,763,862  Total: US$ 231,010,998 | ILI |
| Haas 2014^34^  2012–2013 | Germany | General population | Age NR | **Cost of incapacity to work for 2012/2013 influenza season: € 683,972,690** | ILI |
| Gianino 2019^44^  2010–2013 | Italy |  |  | **Cost for days lost per year** | ILI |
|  |  | HCWs | All ages | € 1,763,683 |  |
|  |  |  | ≥39 | € 445,070 |  |
|  |  |  | 40–49 | € 648,965 |  |
|  |  |  | 50–59 | € 607,107 |  |
|  |  |  | ≥59 | € 84,528 |  |
|  |  | All job categories | All ages | € 1,763,683 |  |
|  |  | Medical doctors |  | € 146,479 |  |
|  |  | Technical executives |  | € 36,984 |  |
|  |  | Nurses and allied health professionals |  | € 794,809 |  |
|  |  | Other executives |  | € 4,180 |  |
|  |  | Nonmedical support staff |  | € 567,584 |  |
|  |  | Administrative staff |  | € 213,648 |  |
| Sendi 2020^45^  2017 |  | HCWs | Age NR | **Total cost across HCWs who had work absence** (n=33)  CHF 87,000  € 81,308  USD 85,294 |  |

Key: CHF – Swiss Franc; CV – cardiovascular; HCW – healthcare worker; ILI – influenza-like illness; LCI – laboratory-confirmed influenza; NR – not reported; SD – standard deviation; US – United States; US$ – United States dollar.

Table S7. Workdays lost

| **Citation**  **Time period** | **Country** | **Risk status** | **Age group (years)** | **Workdays lost** | **Influenza definition** |
| --- | --- | --- | --- | --- | --- |
| **Proportion of employees absent due to influenza** | | | | | |
| Schanzer 2011^40^  1997–2009 | Canada | General population | ≥15 | **Employees absent due to influenza per period (wave/season):** 11.5% | ILI |
| Alberti 2010^37^  2007–2009 | Spain | General population | ≥16 | **Incidence of SL due to influenza:** 915.2–2,377.2 per 100,000 workers | ILI |
| **Proportion of absences attributable to influenza** | | | | | |
| Amodio 2010^43^  2008 | Italy | HCW | Age NR | 11.9% | ILI |
| **Proportion of hours lost annually attributable to seasonal influenza** | | | | | |
| Schanzer 2011^40^  1997–2009 | Canada | General population | ≥15 | 3% | ILI |
| **Proportion of potential working hours that were lost due to own influenza^a^** | | | | | |
| Schanzer 2011^40^  1997–2009 | Canada | General population | ≥15 | 0.08% | ILI |
| **Missed work hours (not conditional on influenza infection)** | | | | | |
| Puig-Asensio 2019^46^ | US | HCW | ≥18 | **Median hours lost per worker due to sick leave during influenza season:** 12 | ILI |
| **Proportion of influenza patients taking sick leave or absent from work** | | | | | |
| Silva 2014^22^  2010–2011 | France |  |  | **Sick leave among patients with influenza B presenting to GP** | LCI |
|  |  | General population | 0–84 | 13% |  |
|  |  |  | 15–64 | 59% |  |
| Scholz 2019^23^  2012–2014 | Germany | General population | All ages | 33.4% | ILI |
| Haas 2014^34^  2012–2013 | Germany | General population | NR | **Incapacity to work caused by influenza:** 35.6% | ILI |
| Ehlken 2015^15^  2010–2012 | Germany | General population | ≥17 | **Influenza episodes with sickness certificate delivered:** 60.7% | ILI |
| Bilcke 2014^21^  2012 | Belgium | General population | All ages | **Respondents reporting interruption of daily activity**  Community patients: 34%  Ambulatory patients: 72%  Hospitalized patients: 88% | ILI |
| Karve 2013a^17^  2002–2009 | US | General population | Age NR | **Patients with an influenza diagnosis who had at least 1 day of influenza-related workplace absence:** 30.1% | ILI |
| Karve 2013c^18^  2008–2009 | US |  |  | **Patients with at least 1 day of influenza-related workplace absence** | ILI |
|  |  | General population | All ages | 30.08%–36.94% |  |
|  |  |  | 18–24 | 22.22%–27.50% |  |
|  |  |  | 25–44 | 29.13%–35.32% |  |
|  |  |  | 45–64 | 30.82%–39.75% |  |
|  |  | Asthma | Age NR | 36.02%–39.46% |  |
|  |  | Diabetes | Age NR | 31.76%–37.1% |  |
|  |  | CV disease | Age NR | 32.65%–38.89% |  |
|  |  | Chronic lung disease | Age NR | 29.00%–37.88% |  |
| **Amount of work lost per employee** | | | | | |
| *Proportion of reduction of productivity due to influenza* | | | | | |
| Van Wormer 2017^47^  2012–2016 | US | General population | ≥18 | **Expected work hours lost per patients since illness onset**  (7–17 days after onset): 67% | LCI |
| *Average amount of work loss due to influenza infection among patients with a sick leave* | | | | | |
| Silva 2014^22^  2010–2011 | France |  |  | **Sick leave duration among patients with influenza B presenting to GP** | LCI |
|  |  | General population | 0–84 | 6.5 days |  |
|  |  |  | 15–64 | 6.5 days |  |
| Petrie 2016^38^  2012–2013 | US |  |  | **Median number of missed work hours during illness** | LCI |
|  |  | General population | 18–83 | All patients: 20.5 hours  Vaccinated: 24 hours  Unvaccinated: 20 hours |  |
| Scholz 2019^23^  2012–2014 | Germany |  |  | **Duration of sick leave among patients who took sick leave** | ILI |
|  |  | General population | All ages | 6.71 days |  |
|  |  |  | 18–34 | 5.63 days |  |
|  |  |  | ≥60 | 8.25 days |  |
| Haas 2014^34^  2012–2013 | Germany | General population | Age NR | **Mean duration of incapacity to work:** 8.15 days | ILI |
| Ehlken 2015^15^  2010–2012 | Germany | General population | ≥17 | 6.0 days | ILI |
| Alberti 2010^37^  2007–2009 | Spain |  |  | **Mean duration of sick leave, among workers with sick leave due to influenza** | ILI |
|  |  | General population | ≥16 | 6.6–7.1 days |  |
|  |  |  | 16–24 | 5.8–5.9 days |  |
|  |  |  | 25–34 | 5.9–6.5 days |  |
|  |  |  | 35–44 | 6.5–7.2 days |  |
|  |  |  | 45–54 | 7.5–8.1 days |  |
|  |  |  | ≥55 | 9–10.7 days |  |
| Tsai 2014^39^  2007–2009 | US |  |  | **Mean work-loss hours per ILI episode** | ILI |
|  |  | General population | 18–34 | 21.4–21.6 hours |  |
|  |  |  | 35–44 | 22.4–23.5 hours |  |
|  |  |  | 45–54 | 23.8–25.7 hours |  |
|  |  |  | 55–64 | 24.1–25.2 hours |  |
| Bilcke 2014^21^  2012 | Belgium | General population | Age NR | Community: 2 days  Ambulatory: 4 days  Hospitalized: 2 days | ILI |
| Xue 2010^36^  2005–2006 | Norway | General population | Age NR | 7.2 days |  |
| Schanzer 2011^40^  1997–2009 | Canada | General population | Age NR | **Hours off per absence:** 14 hours (approximately 20 days per 100 full-time employees during a typical influenza season) | ILI |
| Amodio 2010^43^  2008 | Italy | HCW | Age NR | 4.6 days | ILI |
| *Average amount of work loss due to influenza infection regardless of sick leave* | | | | | |
| Karve 2013a^17^  2009–2009 | US | General population | Age NR | Matched season: 5.12 hours (0.64 days)  Unmatched season: 6.79 hours (0.85 days) | ILI |
| Nichol 2009^41^  2006–2007 | US | General population | 50–64 | All patients: 1.49 days  Vaccinated: 1.41 days  Unvaccinated: 1.72 days | ILI |
| Ehlken 2015^15^  2010–2012 | Germany | General population | ≥17 | 3.3 days | ILI |
| Garattini 2011^42^  2008–2009 | Italy | General population | 24–64 | Vaccinated: 1.23 days  Unvaccinated: 2.64 days | ILI |
| Karve 2013c^18^  2008–2009 | US | General population | All ages: General population | 6.5–7.3 hours (0.8–0.9 days) | ILI |
|  |  |  | 18–24 | 2.6–4.10 hours (0.3–0.5 days) |  |
|  |  |  | 25–44 | 5.2–6.6 hours (0.6–0.8 days) |  |
|  |  |  | 45–64 | 7.6–9.7 hours (1.0–1.2 days) |  |
|  |  | Asthma | All ages | 7.1–12.3 hours (0.9–1.5 days) |  |
|  |  | Diabetes | All ages | 7.5–9.6 hours (0.8–1.2 days) |  |
|  |  | CV disease | All ages | 7.8–15.9 hours (1.0–2.0 days) |  |
|  |  | Chronic lung disease | All ages | 7.2–12 hours (0.9–1.5 days) |  |
| Molinari 2007^25^  2001–2003 | US | General population | 18–49: General population | Outpatient: 1 day  Inpatient: 12 days | ILI |
|  |  |  | 50–64: General population | Outpatient: 2 days  Inpatient: 13 days |  |
|  |  | At-risk | 18–49 | Outpatient: 2 days  Inpatient: 21 days |  |
|  |  |  | 50–64 | Outpatient: 4 days  Inpatient: 24 days |  |
| Gianino 2017^44^  2010–2013 | Italy |  |  | **During epidemic period per profession** | ILI |
|  |  | HCW, medical doctor | Age NR | 1.04 days |  |
|  |  | HCW, technical executive |  | 1.91 days |  |
|  |  | HCW, nurses and allied health professionals |  | 4.7 days |  |
|  |  | HCW, other executive |  | 0.91 days |  |
|  |  | HCW, non-medical support staff |  | 8.57 days |  |
|  |  | HCW, administrative staff |  | 5.22 days |  |
| Sendi 2020^45^  2017 | Sweden |  |  | **Per employee of rehabilitation center with influenza outbreak** | ILI |
|  |  | HCW, nurse care | Age NR | 4.9 days |  |
|  |  | HCW, hotel sector, kitchen, laundry |  | 7 days |  |
|  |  | HCW, physiotherapy, occupational therapy |  | 10 days |  |
|  |  | HCW, social service employee |  | 8 days |  |
|  |  | HCW, others |  | 4.3 days |  |
|  |  | HCW, total |  | 5.3 days |  |
| **Days working while ill** | | | | | |
| Nichol 2009^41^  2006–2007 | US | General population | 50–64 | All patients: 4.39 days  Vaccinated: 3.93 days  Unvaccinated: 5.63 days | ILI |
| **Days of productivity lost per year** | | | | | |
| Molinari 2007^25^  2001–2003 | US |  |  | **Mean days lost based on total people with influenza, annually** | ILI |
|  |  | General population | 18–49 | 10,178,000 days for 8,903,764 patients annually |  |
|  |  |  | 50–64 | 6,616,000 days for 3,098,284 patients annually |  |
|  |  |  | ≥65 | 15,215,000 days for 3,232,826 patients annually |  |

^a^ Time off for “own influenza” refers to workplace absence for a patient’s illness as opposed to that for caring for another person with influenza.

Key: CV – cardiovascular; GP – general practitioner; HCW – healthcare worker; ILI – influenza-like illness; LCI – laboratory-confirmed influenza; NR – not reported; US – United States.

Table S8. Total costs

| **Citation**  **Time period** | **Country** | **Risk status** | **Age group (years)** | **Total costs** | **Influenza definition** |
| --- | --- | --- | --- | --- | --- |
| **Individual cost** | | | | | |
| *Any influenza case* | | | | | |
| Silva 2014^22^  2010–2011 | France |  |  | **Mean cost per influenza B case from French health insurance perspective (indirect cost since fourth day of absence)** | LCI |
|  |  | General population | All ages | € 71.8 |  |
|  |  |  | 15–64 | € 126.1 |  |
|  |  |  | ≥64 | € 41.8 |  |
| Ehlken 2015^15^  2010–2012 | Germany |  |  | **Total cost per episode from societal perspective** | ILI |
|  |  | General population | ≥17 | Mean: € 514  Median: € 421 |  |
|  |  |  | 17–59 | Mean: € 584 |  |
|  |  |  | ≥60 | Mean: € 248 |  |
| Dal Negro 2018^14^  March 2017 | Italy | General population | Age NR | Total cost per episode of influenza or influenza like syndrome  NHS: € 38.71±8.39, family perspective: € 140.33±6.14, including:   - Vaccination, NHS: € 2.46±0.14; family perspective: € 1.81±0.12 - Workdays lost, NHS: NA; family perspective: € 123.52±5.67 - Resource consumption, NHS: € 27.81±7.92; family perspective: NA - Drugs, NHS: € 8.44±0.40; family perspective: € 15.01±0.60 | ILI |
| **Aggregated costs** | | | | | |
| Molinari 2007^25^  2001–2003 | US |  |  | **Total economic burden** | ILI |
|  |  | General population | All ages | US$ 87,067.3 million |  |
|  |  |  | 18–49 | US$ 8,793.9 million |  |
|  |  |  | 50–64 | US$ 18,474.7 million |  |
|  |  |  | ≥65 | US$ 56,054.7 million |  |
| Young-Xu 2017^26^  2010–2014 | US |  |  | **Total economic burden** | ILI |
|  |  | General population (VA patients) | ≥18 | US$ 1,178 billion |  |
|  |  |  | 18–49 | US$ 309 million |  |
|  |  |  | 50–64 | US$ 810 million |  |
|  |  |  | ≥65 | US$ 59 million |  |
| Ehlken 2015^15^  2010–2012 | Germany | General population | ≥17 | **Annual economic burden of physician-attended seasonal influenza** (calculated by multiplying the number of influenza-associated excess consultations by total cost per episode): € 0.2–€ 3.1 billion | ILI |

Key: ILI – influenza-like illness; LCI – laboratory-confirmed influenza; NA – not applicable; NHS – National Health Service; NR – not reported; US – United States; US$ -- United States dollar.
